# Heart Failure Is a Poor Prognosis Risk Factor in Patients Undergoing Cholecystectomy: Results from a Spanish Data-Based Analysis

**DOI:** 10.3390/jcm10081731

**Published:** 2021-04-16

**Authors:** Javier Marco-Martínez, Francisco Javier Elola-Somoza, Cristina Fernández-Pérez, José Luis Bernal-Sobrino, Francisco Javier Azaña-Gómez, José Luis García-Klepizg, Emmanuel Andrès, Antonio Zapatero-Gaviria, Raquel Barba-Martin, Elpidio Calvo-Manuel, Jesus Canora-Lebrato, Noel Lorenzo-Villalba, Manuel Méndez-Bailón

**Affiliations:** 1 Internal Medicine Department, Hospital Clínico San Carlos, Instituto de Investigación Sanitaria del Hospital Clínico San Carlos IdISSC, Universidad Complutense, 28040 Madrid, Spain; javiermarco.z@gmail.com (J.M.-M.); javier.azana@gmail.com (F.J.A.-G.); jgcuenca85@gmail.com (J.L.G.-K.); ecalvo@ucm.es (E.C.-M.); manuelmenba@hotmail.com (M.M.-B.); 2 Fundación Para la Mejora de la Asistencia Sanitaria, 28008 Madrid, Spain; javier.elola@imasfundacion.es (F.J.E.-S.); cristina.fernandez@imasfundacion.com (C.F.-P.); 3 Servicio de Medicina Preventiva, Complejo Hospitalario Universitario de Santiago de Compostela, Universidad de Santiago de Compostela, Rúa da Choupana, s/n, 15706 Santiago, Spain; 4 Servicio de Control de Gestión, Hospital 12 de Octubre, 28041 Madrid, Spain; jluis.bernal@movistar.es; 5 Service de Médecine Interne, Diabète et Maladies Métaboliques, Hôpitaux Universitaires de Strasbourg, 67000 Strasbourg, France; emmanuel.andres@chru-strasbourg.fr; 6 Internal Medicine Department, Hospital Universitario de Fuenlabrada, Facultad de Ciencias de la Salud, Universidad Rey Juan Carlos, Alcorcón, 28942 Madrid, Spain; antonio.zapatero@salud.madrid.org (A.Z.-G.); jesuscanoralebrato@gmail.com (J.C.-L.); 7 Internal Medicine Department, Hospital Universitario Rey Juan Carlos, Facultad de Ciencias de la Salud, Alcorcón, Universidad Rey Juan Carlos, 28933 Madrid, Spain; raquel.barba@hospitalreyjuancarlos.es

**Keywords:** heart failure, cholecystectomy surgery, in-hospital outcomes, length of stay

## Abstract

Background: The incidence of cholecystectomy is increasing as the result of the aging worldwide. Our aim was to determine the influence of heart failure on in-hospital outcomes in patients undergoing cholecystectomy in the Spanish National Health System (SNHS). Methods: We conducted a retrospective study using the Spanish National Hospital Discharge Database. Patients older than 17 years undergoing cholecystectomy in the period 2007–2015 were included. Demographic and administrative variables related to patients’ diseases as well as procedures were collected. Results: 478,111 episodes of cholecystectomy were identified according to the data from SNHS hospitals in the period evaluated. From all the episodes, 3357 (0.7%) were excluded, as the result the sample was represented by 474,754 episodes. Mean age was 58.3 (+16.5) years, and 287,734 (60.5%) were women (*p* < 0.001). A primary or secondary diagnosis of HF was identified in 4244 (0.89%) (*p* < 0.001) and mean age was 76.5 (+9.6) years. A higher incidence of all main complications studied was observed in the HF group (*p* < 0.001), except stroke (*p* = 0.753). Unadjusted in-hospital mortality was 1.1%, 12.9% in the group with HF versus 1% in the non HF group (*p* < 0.001). Average length of hospital stay was 5.4 (+8.9) days, and was higher in patients with HF (16.2 + 17.7 vs. 5.3 + 8.8; *p* < 0.001). Risk-adjusted in-hospital mortality models’ discrimination was high in both cases, with AUROC values = 0.963 (0.960–0.965) in the APRG-DRG model and AUROC = 0.965 (0.962–0.968) in the CMS adapted model. Median odds ratio (MOR) was high (1.538 and 1.533, respectively), stating an important variability of risk-adjusted outcomes among hospitals. Conclusions: The presence of HF during admission increases in hospital mortality and lengthens the hospital stay in patients undergoing cholecystectomy. However, mortality and hospital stay have significantly decreased during the study period in both groups (HF and non HF patients).

## 1. Introduction

Acute cholescystitis (AC) is a frequent complication of biliary tract disease. The development of AC has been reported in approximately 6–11% of patients with a previous history of biliary colic followed up during 7–11 [1]. However, the clinical course of asymptomatic gallstones has not been properly evaluated as data available come from small sample sizes studies or are based on oral cholecystography. In fact, it is known that females are more prone to develop symptomatic diseases compare with males. In males, conservative treatment should be considered if asymptomatic stones are discovered [2]. Cost-effectiveness analyses have not shown advantages of cholecystectomy on life expectancy in patients with asymptomatic cholelithiasis.

It has been noted that 10–15% of the Western population develops gallstones and symptomatic disease is encountered in 1–3% [1]. Treatment of AC is still controversial in terms of the optimal moment for surgical intervention, choice of surgical procedure, or ideal antibiotic treatment in spite of the data available in current medical literature [2]. Currently, in the absence of contraindications, the recommended surgical procedure in the treatment of AC is laparoscopic or closed cholecystectomy [3].

In developed countries, the technological development, the aging of the population with subsequent chronic diseases, and improved access to health care have contributed to the increase in the number of surgical procedures [4]. Surgery has progressively improved in recent years resulting in lower bleeding rates and surgical time. Lee et al. conducted a study evaluating the cardiac complications after noncardiac surgery. They identified six independent predictors of complications that were included in a Revised Cardiac Risk Index (high-risk type of surgery, history of ischemic heart disease, history of congestive heart failure, history of cerebrovascular disease, preoperative treatment with insulin, and preoperative serum creatinine >2.0 mg/dL) [5].

In Spain, 78 surgical procedures/1000 inhabitants are performed, 46% as ambulatory procedures [5]. General surgery together with orthopedic and trauma surgery are the main surgical departments admitting patients in Spain. Cholecystectomy, either scheduled or urgent, open or closed, is the most frequently performed procedure within this group of surgeries and it one of the most frequently procedures in General Service Departments in Spain [6].

Cholecystectomy is subject to perioperative complications during hospitalization. These may be related to the individual patient (comorbidities), the surgical technique, anesthesia, or postoperative care [7]. The most common complications are cardiovascular, respiratory, or infectious [8]. The most frequent cardiovascular complication seen is heart failure (HF), a condition with increasing prevalence in the elderly [9]. The prevalence of HF depends on its definition, but it varies from 1 to 2% to more than 10% among people aged 70 and over in developed countries [10]. The incidence of perioperative complications (including HF) in patients undergoing cholecystectomy doubles in patients undergoing urgent cholecystectomy compared to scheduled procedures, especially in subjects with previous HF, reaching an incidence greater than 4% [11].

This study was aimed at evaluating the influence of heart failure on in-hospital mortality (IHM) and length of stay (LOS) after laparoscopic or closed cholecystectomy and to analyze the incidence of HF in patients undergoing cholecystectomy.

## 2. Methods

### 2.1. Study Design, Data Source and Patient Population

A retrospective observational study of admissions undergoing cholecystectomy was performed. The analysis of our data was obtained from the Minimum Basic Data Set (MBDS) belonging to the National Health System (NHS). Data extraction is carried out through a request to the Ministry of Health and Consumption of the Spanish National Health System. All hospitals in our country are urged to provide with the medical activity data of each patient admitted. For each patient, clinical data such as age, sex, main and secondary diagnosis, procedures performed during hospitalization, discharge destination (including exitus) and early readmission, as well as the average length of stay are collected. In the case of this research we have used the ICD 9 classification for disease codes and procedures.

The hospital admissions analyzed considered all subjects aged over 17 years, discharged in the period 1 January 2007 and 31 December 2015, with a main diagnosis of cholelithiasis (574) who had an open or laparoscopic cholecystectomy procedure (51.22 and 51.23, respectively). All episodes that lacked information about sex, age, date of admission, principal diagnosis, and those classified as major diagnostic category 14 (pregnancy, delivery, puerperium) in relation to the Patient Readjusted Diagnosis Related Groups (APR-DRG) were excluded. This patient classification system groups hospitalization episodes according to principal diagnosis, degree of illness severity and mortality risk.

According to the presence or not of heart failure (HF) as a secondary diagnosis, the selected admission episodes were subsequently divided in two groups. We used to identify HF the following codes: 402.01, 402.11, 402.91, 404.01, 404.03, 404.11, 404.13, 404.91, 404.93 or 420. The complications evaluated in our analysis are detailed in supplementary material (Table S1).

### 2.2. Statistical Analysis

Length of stay (LOS) and in-hospital mortality (IHM) were considered as outcome variables. A multifactorial risk adjustment model taking into consideration clinical and demographic variables of hospital admissions (adjusted for the specific effect of each hospital center) was used as the mortality risk during hospitalization was considered to be the result from individual causes and the quality of care. We used the APR-DRG Mortality Risk Level (RML) and the Centers for Medicare and Medicaid Services (CMS) RML models. The first model (APR-DRG) encompasses independent variables such as age, sex, RML, length of stay (LOS) and heart failure. The CMS model encompasses the 30-day risk-adjusted mortality CMS variables for HF, adapting the data model to the MBDS characteristics and grouping secondary diagnoses following the Clinical Condition Categories (CC) proposed by Pope et al. updated annually by the Agency for Healthcare Research and Quality (AHRQ). The variable included in the adjustments models were obtained through a backward elimination technique. *p* < 0.05 and *p* ≥ 0.10 were set as the significance levels for selection and elimination of risk factors. Model discrimination was evaluated through the receiver operating characteristic (ROC) curve.

Risk-standardized in-hospital mortality ratios (RSMR) were calculated as the ratio of the number of predicted in-hospital deaths based on the hospital’s performance with its observed case mix to the number of expected in-hospital deaths based on the performance of all hospitals with that hospital’s case mix, multiplied by the unadjusted in-hospital mortality of all hospitals. Consequently, if the in-hospital mortality ratio of a specific hospital is higher than the crude mortality rate, then the probability of mortality at that hospital is above the average rate of the hospitals studied.

To evaluate the influence of HF on in-hospital mortality and to control for patient selection bias between patients with and without HF, Propensity Score Matching (PSM) of the CMS risk-adjusted model was used, with the k nearest neighbors option. The mean effect on treated patients (ATT) and 95% confidence intervals were obtained using a caliber of 0.05 without replacement.

Considering the right-skewed nature of the distribution, a Poisson regression model was used to adjust the LOS, considering patient age, sex, and APR-DRG disease severity as risk factors. The expected average LOS was calculated from the individual predictions resulting from the adjusted model. The risk-standardized LOS rate (RSLR) was calculated as the ratio of the average observed to expected LOS. Temporal trends in RSMR and RSLR during the study period were assessed using a Poisson regression model, with year as the only independent variable. In all models, the incidence rate ratio (IRR) was calculated with 95% confidence intervals. Episodes from 2015 were used to analyze the variability of RSMR and RSLR by center characteristics, according to the number of beds.

Variables were expressed as numbers and percentages for qualitative and as mean (SD) for quantitative. Spearman’s rank correlation coefficient (*p*) was used to analyze the correlation between continuous variables. To compare two categories of quantitative variables we used Student’s t test and ANOVA corrected by the Bonferroni test to compare three or more categories. The 2’s test or Fisher’s exact test was used to compare categorical variables. All statistical contrasts were bilateral and differences were considered significant for *p* < 0.05. Statistical analysis was carried out through STATA 13 and SPSS 21.0.

## 3. Results

In the study period, we found 478,111 episodes with a procedure code of cholecystectomy discharged from SNHS hospitals. The study population was represented by 474,754 episodes of care as 3357 (11.02%) did not meet the inclusion criteria (not mutually exclusive); 2631 (0.55%) were under 18 years old; 867 (0.18%) lacked information about sex, age, admission date, or principal diagnosis; and 192 (0.04%) were classified as major diagnostic category 14. General characteristics are shown in Table 1.

Mean age was 58.3 (+16.5) years, and most were female (287,734 (60.5%), *p* < 0.001). In this population, 4244 (0.89%) had a main or secondary diagnosis of HF at a mean age 76.5 (+9.6) years, significantly older than the rest. Major complications were more common in patients with HF except for stroke. All-hospitals unadjusted IHM was 1.1%, especially in patients with HF (12.9%) versus to those without (1%). Average LOS (5.4 days (+8.9)) was significantly higher in the group with HF (16.2 + 17.7 vs. 5.3 + 8.8; *p* < 0.001).

Risk-adjusted in-hospital mortality models are displayed in Table 2 and Table 3.

**Figure 1:**
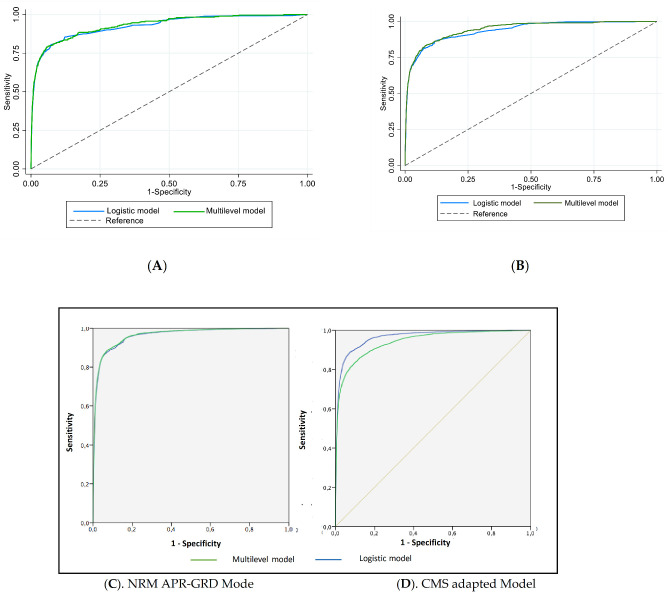
ROC curves of in-hospital mortality risk adjustment (**A**,**B**). APR-DRG RML and CMS models (**C**,**D**).

ROC curves are shown in Figure 1. Discrimination was high in both cases, with AUROC values = 0.963 (0.960–0.965) in the APRG-DRG model and AUROC = 0.965 (0.962–0.968) in the CMS adapted model. Median Odds ratio (MOR) was high (1.538 and 1.533, respectively), showing that the variability between hospitals of risk-adjusted outcomes was high.

After including HF as an independent variable in the risk-adjusted models, both showed a significantly high association with in-hospital mortality OR = 1.31 (1.16–1.48) in the NRM APR-GRD model and OR = 1.758 (1.559–1.983) in the CMS adapted model (*p* < 0.001 in both cases). ATT estimated from PSM was statistically significant (*p* = 0.063) in episodes presenting HF and 0.05 in those who did not (*p* = 0.38), with a relative risk (RR) of 1.27. Unadjusted in-hospital mortality was 1.1%, 12.9% in the group with HF versus 1% in the non HF group (*p* < 0.001). There were no statistically significant differences in in-hospital mortality risk in HF patients between open (1.33; CI: 1.15–1.53) and laparoscopic cholecystectomy procedures (1.35; CI: 1.05–1.74).

The adjusted model for LOS according to APR-GRD severity levels is shown in Table 4. Patients with HF had a significantly longer average LOS (18.08 + 10.27 vs. 5.3 + 4.4; *p* < 0.001), but no significant differences were found between the respective RSLR (0.981 + 1.319 vs. 0.99 + 1.214; *p* = 0.456).

During the study period, the incidence of cholecystectomy procedures increased annually, from 46,823 to 55,430 episodes without HF, and from 400 to 594 with HF. No correlation was found between the volume of episodes per center and the RSMR and the development of HF (*p* > 0.05). In-hospital mortality decreased in both episodes without HF (IRR = 0.9896; *p* = 0.037) and episodes with HF (IRR = 0.9642; *p* = 0.039). Average LOS also decreased yearly from 6.55 + 10.12 (2007) to 4.56 + 8.49 days (2015), decreasing from 6.45 + 9.96 (2007) to 4.47 + 8.03 (2015) days in the group without HF and 18.16 + 18.7 (2007) to 14.43 + 17.84 (2015) days in the HF group). The RSLR decreased 4.92% per year (IRR = 0.9508) both in the HF group (IRR = 0.9872) and the non-HF group (IRR = 0.9505) (*p* < 0.001 in all cases).

In episodes of care with HF in 2015, no significant differences were found between mean RSMR by hospital type, ranging from 11.86 (+2.19) in type 1 hospitals (<100 beds) to 13.53 (+5.15) in type 4 hospitals (>1000 beds) (*p* > 0.05). Likewise, there were no significant differences in RSLR (*p* = 0.43).

## 4. Discussion

We have analyzed a large clinical–administrative database with more than 470,000 cholecystectomies in all hospitals in Spain over a period of 9 years in order to identify heart failure (HF) and its impact on patients undergoing this surgical procedure. Among the patients with HF, we found more women, in accordance with the population of patients undergoing surgical procedure at an older age [12]. The low incidence of HF in our series is surprising, something that may be related to the low coding in discharge reports, a problem already reported when working with large clinical–administrative databases [6,13].The mean age of the series is slightly higher compared to others found in the literature [7,14].

Patients with HF are significantly older for several reasons. Age is one major risk factor in the development of HF [12]. A preponderance of men in the group with HF, as seen in this study, is expected given the differences in epidemiology of HF between sexes [13]. It is more common for the origin of heart disease in men to be ischemic, and it is possible that some of them could have died before they reached the mean age of our series or their surgical risk was too high to undergo an intervention. We observed a higher mean age in the group of patients with HF, something to be expected and already described in different investigations reporting an increase in the risk of perioperative HF with age [14].

All the comorbidities analyzed, except ischemic stroke, were more frequently encountered in the HF group. Acute myocardial infarction (AMI), acute pulmonary edema (APE), and cardiogenic shock are directly related to the underlying pathophysiology of HF and their relationship is therefore not surprising. This was not the case with ischemic stroke although it is certainly an event associated with HF [2]. The most frequent comorbidities found in our series were peripheral vascular disease, hypertension, DM, dementia, pneumonia, kidney failure, heart failure, arteriosclerosis, and COPD. In addition, they were all significantly more frequent in the population with HF. Risk adjustment models for in-hospital mortality achieved good sensitivity and specificity results both by adjusting for patient severity and by considering inter-hospital variability. The same is achieved in the case of hospital stay. For both variables, the presence of HF during admission increases with risk of mortality and lengthens the hospital stay [13]. The risk of complications is mainly related to the patient’s comorbidities, while factors such as the hospital structure or the surgeon’s expertise are of less importance [13]. As age increases, the presence of comorbidities and frailty increases and therefore the risk of complications [7,13].

Certainly the development of a complication such as HF during hospitalization for a surgical process lengthens the stay; our study found nearly a three-fold increase in risk. There are studies in the medical literature about HF complicating surgical procedures, especially cardiac surgery [13] and cholecystectomy, most of which focused on the development of predictive models of mortality [14]. Most of these models considered factors such as age, some comorbidities, or analytical data to try to estimate the risk of death after the surgical procedure [8].

In our series, the crude mortality rate was 1.1%, significantly higher among patients who presented with HF (12.9%) than among those who did not (1%) (*p* < 0.001). Previous studies have also demonstrated a strong association in patients with heart failure or dilated cardiomyopathy, whose risk of death is 3.6 times higher than that of patients for whom no such comorbidity is coded [15,16].

The increase in hospital mortality related to HF has been well described [13]. Comorbid conditions and functional status play a key role in elderly individuals which has been associated with the concept of frailty. Regarding the total number of procedures performed during our study period, we noted a consistent increase in patients with and without HF. This was an expected phenomenon influenced by development of health service in our country and the rest of the world [13].

We have identified a statistically significant decrease in hospital mortality during the study period in patients with and without HF. The better results in in-hospital mortality during cholecystectomy in our country could be attributed to a better planning of the surgery and the time chose for the surgery. In our environment, less and less urgent surgeries are performed and there is a tendency to privilege antibiotics in elderly patients and perform surgery when the clinical situation stable Besides, less invasive procedures such as laparoscopic surgery are preferred. In recent years, internists have begun to work for surgical specialists as consultants and manage the perioperative period of increasingly multimorbid and frail patients before surgical procedures that grow in complexity and aggressiveness. Interconsultation units have been incorporated into the activities of internists in the last 10 years in support of surgical specialists [17].

This study, based on data obtained from a clinical-administrative database, has some limitations related to insufficient information such as: (1) lack of data about the moment in which HF appears, if the patient developed HF during admission or if it was a comorbid condition and at what point in the postoperative period HF appeared; (2) if re-admission occurred after the surgical procedure; (3) pharmacological treatment; (4) analytical data.

Another limitation is undoubtedly dependent on a discharge report made by physicians who may or may not code all of the patients’ diagnoses and procedures. The under-coding of HF that possibly exists in our study may reflect a defect in the diagnosis, a problem in the coding, or a truly low prevalence. The relative absence of the phenotype in these large databases due to under-coding could limit the usefulness of predictive models based on this type of information. On the other hand, this large data base gives us significant statistical power and the possibility of analyzing a long period of time and an extensive geographical territory.

## 5. Conclusions

The presence of HF during admission increases in hospital mortality and lengthens the hospital stay in patients undergoing cholecystectomy. However, mortality and hospital stay have significantly decreased during the study period in both groups (HF and non HF patients).

## Figures and Tables

**Table 1:** General characteristics according to the presence of heart failure.

	Group without HF		Group with HF		Total		*p*
	N	%	N	%	N	%	
Women	284,734	60.5	2273	53.6	287,007	60.5	<0.001
>64 years old	188,321	40	3803	89.6	192,124	40.5	<0.001
History of PTCA	5017	1.1	123	2.9	5140	1.1	<0.001
History of CABG	2327	0.5	65	1.5	2392	0.5	<0.001
Congestive heart failure	4123	0.9	4239	99.9	8362	1.8	<0.001
Acute myocardial infarction	195	0.04	62	1.46	257	0.1	<0.001
Stroke	11	0	0	0	11	0	0.753
Acute pulmonary edema or cardiogenic shock	4782	1.02	869	20.5	5651	1.2	<0.001
Pulmonary thromboembolism	225	0.05	22	0.52	247	0.1	<0.001
Other Acute/subacute forms of ischemic heart disease	463	0.1	95	2.2	558	0.1	<0.001
Chronic atherosclerosis	17,842	3.8	629	14.8	18,471	3.9	<0.001
Cardio-respiratory failure and shock	6757	1.4	1019	24	7776	1.6	<0.001
Valvular and rheumatic heart disease	5717	1.2	549	12.9	6266	1.3	<0.001
Hypertension	152,572	32.4	2391	56.3	154,963	32.6	<0.001
Stroke	256	0.1	21	0.5	277	0.1	<0.001
Renal failure	11,410	2.4	1054	24.8	12,464	2.6	<0.001
COPD	17,599	3.7	614	14.5	18,213	3.8	<0.001
Pneumonia	31,567	6.7	816	19.2	32,383	6.8	<0.001
Diabetes Mellitus and complications	61,148	13	1255	29.6	62,403	13.1	<0.001
Protein-energy malnutrition	1343	0.3	93	2.2	1436	0.3	<0.001
Dementia and senility	14,052	3	312	7.4	14,364	3.0	<0.001
Hemiplegia, paraplegia, paralysis, function disability	3934	0.8	99	2.3	4033	0.8	<0.001
Peripheral vascular disease	160,924	34.2	2893	68.2	163,817	34.5	0.001
Severe cancer	8728	1.9	164	3.9	8892	1.9	<0.001
Trauma	563	0.1	15	0.4	578	0.1	<0.001
Mayor psychiatric disorders	2559	0.5	26	0.6	2585	0.5	0.545
Chronic liver disease	2352	0.5	35	0.8	2387	0.5	0.003

N: number of episodes. PCTA: Percutaneous transluminal coronary angioplasty, CABG: Coronary artery bypass grafting, COPD: Chronic Obstructive Pulmonary Disease.

**Table 2:** Multilevel risk adjustment model of in-hospital mortality according to APR-DRG Risk Mortality Level.

	Odds Ratio	Standard Error	z	*p*	[95% Confidence Interval]	
Men	0.9548	0.0368	−1.2	0.23	0.8854	1.0297
Age	1.0429	0.0019	22.78	<0.001	1.0392	1.0467
Length of stay	1.0028	0.0008	3.52	<0.001	1.0013	1.0044
Risk mortality level						
2	8.1620	0.6371	26.9	<0.001	7.0041	9.5112
3	73.8966	5.5485	57.3	<0.001	63.7841	85.6123
4	450.6372	32.9975	83.45	<0.001	390.3900	520.1821
Intercept	0.0001	0.0000	−63.69	<0.001	0.0000	0.0001

**Table 3:** Multilevel risk adjustment model of in-hospital mortality according to CMS model.

	Odds Ratio	Standard Error	z	*p*	[95% Confidence Interval]	
Age > 64 years	3.7800	0.1934	25.99	<0.001	3.4193	4.1787
Male	0.8804	0.0354	−3.17	0.002	0.8137	0.9526
History of PCTA	0.9560	0.1510	−0.28	0.776	0.7015	1.3029
History of CABG	0.8726	0.1868	−0.64	0.524	0.5736	1.3274
Congestive heart failure	1.7584	0.1079	9.2	0	1.5592	1.9830
Acute myocardial infarction	1.5086	0.4913	1.26	0.207	0.7969	2.8561
Other forms of ischemic heart disease	2.3414	0.5782	3.44	0.001	1.4430	3.7991
Chronic atherosclerosis	1.0430	0.0807	0.54	0.587	0.8961	1.2139
Cardio-respiratory failure and shock	29.2417	1.2661	77.96	0	26.8626	31.8316
Valvular and rheumatic heart disease	1.2085	0.1195	1.92	0.055	0.9957	1.4669
Hypertension	0.4171	0.0311	−11.7	0	0.3605	0.4826
Stroke	15.8388	3.0534	14.33	0	10.8549	23.1109
Renal failure	6.4102	0.2862	41.61	0	5.8730	6.9965
Stroke	0.8630	0.0577	−2.21	0.027	0.7571	0.9837
Pneumonia	1.9177	0.0901	13.86	0	1.7490	2.1026
Diabetes Mellitus and complications	0.9670	0.0473	−0.69	0.493	0.8786	1.0643
Protein-energy malnutrition	1.8103	0.2400	4.48	<0.001	1.3961	2.3473
Dementia	1.4159	0.0977	5.04	<0.001	1.2369	1.6208
Hemiplegia, paraplegia, paralysis, and functional disability	1.7422	0.2151	4.5	<0.001	1.3677	2.2192
Peripheral vascular disease	1.9542	0.1443	9.07	<0.001	1.6908	2.2585
Severe cancers	3.8330	0.2514	20.49	<0.001	3.3706	4.3588
Trauma	4.7790	0.9708	7.7	<0.001	3.2094	7.1161
Major psychiatric disorders	0.8416	0.2144	−0.68	0.499	0.5109	1.3866
Chronic liver disease	3.1831	0.4347	8.48	<0.001	2.4355	4.1601
Intercept	0.0016	0.0001	−74.7	<0.001	0.0014	0.0019

PCTA: Percutaneous transluminal coronary angioplasty, CABG: Coronary artery bypass grafting, COPD: Chronic Obstructive Pulmonary Disease.

**Table 4:** Risk adjustment model of length of stay according to APR-DRG Risk Severity Level.

	IRR	Standard Error	z	*p*	[95% Confidence Interval]	
Men	0.8888	0.0038	−27.75	<0.001	0.8815	0.8963
Age	1.0080	0.0001	53.8	<0.001	1.0077	1.0083
Risk severity level						
2	1.8575	0.0079	146.15	<0.001	1.8422	1.8730
3	4.4397	0.0326	203.22	<0.001	4.3763	4.5040
4	8.3490	0.1056	167.82	<0.001	8.1447	8.5585
Intercept	2.6185	0.0296	85.28	<0.001	2.5612	2.6771

IRR: Incidence Rate Ratio.

## Data Availability

https://www.mdpi.com/ethics (accessed on 12 April 2021).

## Supplementary Materials

The following are available online at https://www.mdpi.com/article/10.3390/jcm10081731/s1, Table S1: Complications studied.

## References

[1] Friedman G.D. (1993). Natural history of asymptomatic and symptomatic gallstones. Am. J. Surg..

[2] Díaz-Gómez D., Parra-Membrives P., Villegas-Portero R., Molina-Linde M., Gómez-Bujedo L., Lacalle-Remigio J.R. (2012). Análisis del tratamiento quirúrgico más apropiado para la colecistitis aguda mediante aplicación del método RAND/UCLA. Cir. Esp..

[3] Bueno Lledó J., Granero Castro P., i Gavara I.G., Ibañez Cirión J.L., López Andújar R., García Granero E. (2016). Twenty-five years of ambulatory laparoscopic cholecystectomy. Cir. Esp..

[4] Provisión de Servicios Sanitarios. http://www.mscbs.gob.es/organizacion/sns/planCalidadSNS/pdf/equidad/Informe_Anual_Anexo_VI.pdf.

[5] Lee T.H., Marcantonio E.R., Mangione C.M., Thomas E.J., Polanczyk C.A., Cook E.F., Sugarbaker D.J., Donaldson M.C., Poss R., Ho K.K.L. (1999). Derivation and Prospective Validation of a Simple Index for Prediction of Cardiac Risk of Major Noncardiac Surgery. Circulation.

[6] Ministerio de Sanidad y Consumo. https://www.mscbs.gob.es/estadEstudios/sanidadDatos/tablas/tabla26.htm.

[7] Giger U.F., Michel J.-M., Opitz I., Inderbitzin D.T., Kocher T., Krähenbühl L. (2006). Risk Factors for Perioperative Complications in Patients Undergoing Laparoscopic Cholecystectomy: Analysis of 22,953 Consecutive Cases from the Swiss Association of Laparoscopic and Thoracoscopic Surgery Database. J. Am. Coll. Surg..

[8] Sato M., Endo K., Harada A., Yabuuchi S. (2017). Potential risk factors for postoperative complications and deaths after laparo-scopic cholecystectomy in the elderly. Nihon Shokakibyo Gakkai Zasshi.

[9] Lauro A., Cervellera M., D’Andrea V., Casella G., Di Matteo F.M., Di Matteo F.M., Santoro A., Panarese A., Palazzini G., Ci-rocchi R. (2019). Impact of cardiovascular/diabetic comorbidity on conversion rate during laparoscopic cholecystectomy for acute cholecystitis: A multi-center study on early versus very delayed ap-proach. G. Chir..

[10] Loehr L.R., Rosamond W.D., Chang P.P., Folsom A.R., Chambless L.E. (2008). Heart failure incidence and survival (from the Athero-sclerosis Risk in Communities study). Am. J. Cardiol..

[11] Hall C.M., Jupiter D.C., Regner J.L. (2016). Newly diagnosed and decompensated congestive heart failure is associated with in-creased rates of pneumonia, reintubation, and death following laparoscopic cholecystectomy: A NSQIP database review of 143,761 patients. Int. J. Surg..

[12] Shahian D.M., Normand S.-L., Torchiana D.F., Lewis S.M., Pastore J.O., Kuntz R.E., Dreyer P.I. (2001). Cardiac surgery report cards: Comprehensive review and statistical critique11This review is an abridged version of a report submitted by the Massachusetts Cardiac Care Quality Commission to the Massachusetts Legislature, May 2001. Ann. Thorac. Surg..

[13] Damrauer S.M., Gaffey A.C., Smith A.D., Fairman R.M., Nguyen L.L. (2015). Comparison of risk factors for length of stay and readmission following lower extremity bypass surgery. J. Vasc. Surg..

[14] Zapatero Gaviria A., Barba Martín R., Román Sánchez P., Casariego Vales E., Diez Manglano J., García Cors M., Jusdado Ruiz-Capillas J.J., Suárez Fernández C., Bernal J.L., Elola Somoza F.J. (2016). RECALMIN. La atención al paciente en las unidades de Medicina Interna del Sistema Nacional de Salud. Rev. Clínica Esp..

[15] Nimptsch U., Mansk T. (2015). Deaths Following Cholecystectomy and Herniotomy: An Analysis of Nationwide German Hospital Discharge Data From 2009 to 2013. Dtsch. Arztebl Int..

[16] Barba R., Marco J., Canora J., Plaza S., Juncos S.N., Hinojosa J., Bailon M.M., Zapatero A. (2015). Prolonged length of stay in hospital-ized internal medicine patients. Eur. J. Intern. Med..

[17] Boehme J., McKinley S., Michael Brunt L., Hunter T.D., Jones D.B., Scott D.J., Schwaitzberg S.D. (2016). Patient comorbidities increase postoperative resource utilization after laparoscopic and open cholecystectomy. Surg. Endosc..

