# Supplementary material for: Heart Failure Is a Poor Prognosis Risk Factor in Patients Undergoing Cholecystectomy: Results from a Spanish Data-Based Analysis
Source: J Clin Med. 2021 Apr 16;10(8):1731. doi: 10.3390/jcm10081731 (PMC8072897; doi:10.3390/jcm10081731)
Supplement: Supplementary file 1 [file jcm-10-01731-s001.zip › jcm-1171521-supplementary.pdf]

Supplementary material

Table S1. Complications studied

Secondary diagnosis	ICD-9-CM code
Acute myocardial infarction	410.*0, 410.*1
Acute pulmonary edema or cardiogenic shock	427.41, 427.42, 427.5, 518.4, 518.5, 518.51, 518.52, 518.53, 518.81, 518.82, 518.83, 518.84,785.50, 785.51, 798.0, 798.1, 798.2, 798.9, 799.01, 799.02, 998.01
Stroke	433.01; 433.11; 433.21; 433.31; 433.81; 433.91; 434.01; 434.11; 434.91; 436)
Pulmonary thromboembolism	415.1, 415.11, 415.12, 415.13, 415.19
